# Concerning the debate about homocysteine, B vitamins, and dementia

**DOI:** 10.1177/13872877251350297

**Published:** 2025-06-23

**Authors:** Joshua W Miller, Andrew McCaddon, Jin-Tai Yu, Babak Hooshmand, Helga Refsum, A David Smith

**Affiliations:** 1 Department of Nutritional Sciences, 57674 Rutgers University, New Brunswick, NJ, USA; 2 Faculty of Social and Life Sciences, 8725 Wrexham University, Wrexham, UK; 3 Department of Neurology, Huashan Hospital, 12478 Fudan University, Shanghai, China; 4 Aging Research Center, 27106 Karolinska Institute, Stockholm, Sweden; 5 Department of Nutrition, University of Oslo, Oslo, Norway; 6 Department of Pharmacology, 6396 University of Oxford, Oxford, UK

**Keywords:** Alzheimer's disease, B6, B12, dementia, folate, homocysteine, risk factor

## Abstract

It is important to identify modifiable risk factors for dementia and to introduce policies to implement their modification. The Lancet Commission on Dementia Prevention, Intervention and Care failed to identify raised plasma homocysteine as a risk factor, despite considerable evidence; hence there is a need for a debate on this matter.

In 2018 we published an ‘International Consensus Statement on Homocysteine and Dementia’ in this journal, in which we concluded that elevated plasma total homocysteine is a modifiable risk factor for development of cognitive decline, dementia, and Alzheimer's disease (AD) in older persons.^1^ We further stated that intervention trials in elderly with cognitive impairment show that homocysteine-lowering treatment with B vitamins markedly slows the rate of whole and regional brain atrophy, and also slows cognitive decline. We were therefore puzzled why the Lancet Commission on dementia prevention, intervention and care failed to discuss a possible role of homocysteine and B vitamins in all three of their reports, including the most recent one.^2^ We submitted a Letter pointing this out but *The Lancet* declined to publish the Letter and sent us a rebuttal from members of the Commission. Our submitted but unpublished Letter appeared on the web site of ‘Food for the Brain’ as one of a group of three Letters concerning the lack of discussion of certain nutritional factors by the Commission.^3^ *The Lancet* has now published a Letter from the Commission^4^ commenting upon our unpublished Letter. We wish to reply to the Commission and continue the debate with the aim of reaching a common view on homocysteine, B vitamins and dementia. This is an important matter of public health.

The Commission authors discuss^4^ the VITACOG trial in which subjects with mild cognitive impairment (MCI) were treated for two years with high doses of folic acid and vitamins B6 and B12.^5^ The trial was designed to see if this treatment slowed the accelerated rate of brain atrophy that occurs in subjects with MCI: the outcome was positive with a 31% reduction in whole brain atrophy rate in the vitamin-treated subjects compared with placebo.^5^ (There was a 30% decrease in plasma total homocysteine concentration in the treated subjects.) A subset of the participants with a baseline plasma homocysteine above the median (11.3 μmol/L) showed cognitive benefits from the treatment and in those with homocysteine in the top quartile there were also clinical benefits, such as improved Clinical Dementia Rating (CDR) score.^6^ The Commission authors comment on a further analysis of regional brain atrophy in VITACOG in which it was found that the rate of atrophy of those brain regions, such as the medial temporal lobe, typically affected in AD was reduced by more than 7-fold by the vitamin treatment in subjects with homocysteine above the median.^7^

The Commission authors interpreted the evidence from the latter paper^7^ cautiously because they considered it was a *post-hoc* analysis. But the analysis of regional brain atrophy is a natural part of the original aim of the trial to study the effect of B vitamin treatment on brain atrophy and was, in fact, included in the original analysis plan. We therefore consider that the results of this paper should be assessed on their merits. Furthermore, we are puzzled by the Commission authors’ statement, “the results do not show benefits in populations already consuming B vitamins in their food or through supplements*”*.^4^ Exclusion criteria for the VITACOG trial included taking folic acid supplements >300 µg/d, pyridoxine supplements >3 mg/d or vitamin B12 supplements >1.5 µg/d by mouth or any dose by injection. It did not exclude individuals taking supplements below these cutoffs (∼16–20% of the participants were taking low dose supplements), nor did it exclude any individuals based on their regular diets, which of course include these B vitamins at various levels.^5^ Notably, the supplement and dietary characteristics of those included in the study are representative of most individuals, including those with MCI, who are at risk of dementia. The comment by the Commission authors seems to suggest that in order to consider the findings meaningful and relevant they should have been obtained in participants who had much better B vitamin intake and status than those included in the study. However, those with better B vitamin intake and status are those we would not expect to benefit from the supplements, as indeed we found, in that those with low homocysteine (likely with good B vitamin status) did not benefit. The Commission authors’ comment is analogous to expecting additional drug treatment to provide benefits over and above the benefits being obtained in people already taking a high dose of the drug, which is why it puzzles us.

The Commission authors cite a trial in Hong Kong^8^ in which there was no effect of B vitamins on cognitive decline in MCI over 2 years, although there were some benefits after 1 year. One reason for this lack of benefit might be because 22% of participants were also taking aspirin, which the authors found impaired the beneficial effect of B vitamins. This finding has since been confirmed.^9^ Another possible reason for lack of benefit is that the participants may have had a low omega-3 fatty acid status (see below under ‘Clinical trials’).

The Commission authors cite the VITAL trial of B vitamins in patients with a diagnosis of AD as showing no cognitive benefit.^10^ However, in a subgroup analysis the authors of the VITAL trial described a significant beneficial effect in the subgroup whose MMSE score was above the median at the start and who had a CDR score of 0.5 (i.e., consistent with MCI or early-stage AD). The authors kindly provided these data, which we showed in Figure 7 in our review article.^11^

We mention this result here because it illustrates an important point: patients with mid-to-late-stage AD may not respond to B vitamins because the disease process has gone too far. As pointed out by Aisen et al.,^10^ this suggests that trials at an earlier stage, like MCI or early-stage AD, might be more successful. Nevertheless, a recent trial of folate and vitamin B12 in patients with a diagnosis of probable AD did show beneficial effects in several areas of cognition.^12^

The Commission authors say, “there is an absence of positive studies showing that vitamin B supplementation mitigates dementia or cognitive decline”. We agree that there is little evidence of an effect in patients with established dementia, possibly for the reason mentioned above, but there is in fact good evidence for an effect on cognitive decline in people with MCI, as we summarize below. (The Commission authors refer to a Cochrane review in 2018;^13^ but we note that at the time one of us published a Comment on the review pointing out the importance of taking the baseline homocysteine concentration into consideration.^14^)

## Brief overview of current knowledge about homocysteine, B vitamins, and cognition

We will not attempt a comprehensive review of this topic in this commentary. Three of our earlier reviews summarized the evidence of a link between homocysteine, B vitamins and cognition.^1,11,15^ With one exception,^16^ several more recent reviews and meta-analyses have confirmed this link and have found a beneficial effect of B vitamins.^17–20^ It is pertinent that, from their review of 243 prospective observational studies and 153 randomized trials, Yu et al.^17^ concluded, “Notably, homocysteine-lowering treatment seems the most promising intervention for AD prevention.”

Here, we would like to mention some key findings.

### Observational studies

Several are covered in the reviews listed above, but we wish to draw attention to a large study using the UK Biobank which covered 192,214 participants whose dietary intake of various factors involved in one-carbon metabolism was assessed in relation to the subsequent outcome of a diagnosis of AD.^21^ We believe it is of particular relevance to the matter under discussion. This study looked at the dietary intake of methionine, folate, and vitamins B6 and B12 in relation to the risk of AD 13y later and found a marked protective effect of the intake of these substances against the development of AD, with hazard ratios for the top quartile of intake versus the bottom quartile ranging from 0.30 to 0.77. Homocysteine is not yet measured in the UK Biobank so was, unfortunately, not included.

The study by Wang et al.^21^ found that the dietary intake of methionine was strongly protective against the development of late-onset AD, with a hazard ratio of 0.3 for the top versus bottom quartile, but blood levels were not measured. A Swedish study measured methionine and homocysteine in blood from a cohort of 2570 elderly people that was followed for 6 years.^22^ Those in the top quartile of blood methionine had a 46% lower risk of dementia than those in the bottom quartile whereas those in the top quartile of homocysteine had a 60% greater risk of dementia compared with those in the bottom quartile. This study also found that the methionine-to-homocysteine ratio was positively related to the blood levels of folate and B12 and that participants with a high methionine-to-homocysteine ratio (indicating a better methylation status) showed a slower rate of both overall brain atrophy and gray matter atrophy over time than those with a low ratio.^22^

Although the most common reason for raised blood homocysteine levels is a dietary deficiency or suboptimal status of B vitamins, several other factors can lead to raised levels,^23^ some of which are also recognized risk factors for dementia. An example is air pollution, which a Swedish study found was associated with a 70% increased dementia risk per unit increase in PM_2.5_ over 5y and that half of the increased risk was related to raised blood homocysteine.^24^ In contrast, higher blood methionine was associated with a reduced risk of dementia from air pollution.^24^ (Notably, reducing exposure to air pollution is one of the factors that the Lancet Commission suggests will reduce risk of dementia.^2^)

#### Clinical trials

There are relatively few clinical trials of homocysteine-lowering treatments. One of the earliest trials is the VITACOG trial^5^ mentioned above. An important conclusion from this trial is that cognitive and clinical benefits only occur in participants with a relatively high homocysteine at baseline. It makes sense that those with low homocysteine, reflecting adequate B vitamin status, would not benefit from further B vitamins. This point should be born in mind when assessing the results of other trials.

In the VITACOG trial, Bayesian network analysis was used to identify causal links and the following pathway was found: *B vitamin treatment lowers homocysteine, which slows grey matter atrophy, which delays worsening of CDR, which slows MMSE decline.*^7^ We have thus argued that the results from the VITACOG trial are consistent with a disease-modifying effect of B vitamins in MCI.^25^

An important *post-hoc* result from VITACOG was the finding that the beneficial effects of B vitamins only occurred in participants who had good omega-3 fatty acid status. This applied both to the slowing of brain atrophy^26^ and the slowing of cognitive decline.^27^ This finding needs to be taken into account when looking at the results of other B vitamin trials. It was found, for example, that in the B-proof trial the apparent lack of cognitive benefits from B vitamins^28^ was because no account had been taken of the status of an omega-3 fatty acid, docosahexanoic acid (DHA). A *post-hoc* study showed that in participants with good DHA status, B vitamin supplements showed cognitive benefits.^29^ A short trial in China compared the effect of folic acid alone and folic acid with DHA in people with MCI and found that the cognitive benefits of the combined treatment were greater than with either nutrient on its own.^30^ It is noteworthy that at a population level there is also an interaction between raised homocysteine, low omega-3 status and the risk of dementia.^31^ This topic has been reviewed.^32^

Other subgroup effects might be important and should be considered when planning or assessing trials. For example, the use of aspirin can impair the beneficial cognitive and brain structural responses to B vitamins.^9^ Another subgroup effect was found when examining the outcomes of trials in relation to a genetic polymorphism in dihydrofolate reductase, the enzyme that reduces folic acid thus making it active in folate-dependent pathways. The beneficial cognitive effect of folic acid-containing B vitamins was only found in the subjects who had the more active variant with the *ins/ins* genotype.^33^ Natural dietary folates are in the reduced form and do not require this enzyme for activation. Future trials might thus consider using reduced folates instead of folic acid to lower homocysteine. Accordingly, we suggest that a new trial should be designed to see if the conversion from MCI to dementia can be slowed or prevented by selecting patients with MCI who have a high plasma total homocysteine (above 11 μmol/L), who are not taking aspirin, and treating them with high doses of a reduced folate, vitamins B6 and B12, together with omega-3 fatty acids.

## Conclusion

We hope that the Lancet Commission will consider the substantial existing evidence of raised homocysteine as an important risk factor for dementia and the possibility of modifying its harm by supplementation with B vitamins. The evidence is as strong or stronger than many of the other modifiable risk factors that the Commission includes in its 2024 publication. Continued dismissal of the potential beneficial effects of B vitamins and homocysteine-lowering in older individuals at risk of dementia represents a major missed opportunity in medicine and public health.
